# Integrated ^64^Cu therapy for the peritoneal dissemination of gastrointestinal cancer

**DOI:** 10.18632/oncotarget.25840

**Published:** 2018-07-27

**Authors:** Hiroaki Kurihara

**Affiliations:** Department of Diagnostic Radiology, National Cancer Center Hospital, Tokyo, Japan

**Keywords:** Cu-64, EGFR, radiotherapy

Peritoneal dissemination is a frequent cause of death in patients with gastrointestinal cancers. It is difficult to treat with conventional therapeutics, particularly when it proceeds to a late phase. Hence, there is a strong need to develop new treatment strategies for peritoneal dissemination, to improve patient survival.

Recently, a Japanese team from the National Institute of Radiological Sciences (National Institutes for Quantum and Radiological Science and Technology), National Cancer Center Hospital East, and Nihon Medi-Physics Co., Ltd. published a study in the journal *Oncotarget* [1] to address this problem.

In this study, Dr. Yoshii and her colleagues developed a novel and invocative treatment strategy, called an “integrated ^64^Cu therapy” that used ^64^Cu-intraperitoneal radioimmunotherapy (ipRIT), alone or in combination with positron emission tomography (PET)-guided surgery to treat early- and late-phase peritoneal dissemination in mouse models. To achieve this new strategy, they focused on a theranostic agent, a ^64^Cu-labeled anti-epidermal growth factor receptor (EGFR) antibody cetuximab. Cetuximab is widely used in clinical practices for a large variety of cancers, because many malignant tumors show EGFR overexpression [2]. The significance of using ^64^Cu-labeled cetuximab is that this probe can be simultaneously used for both imaging and therapy. ^64^Cu is a radionuclide that uniquely emits positrons, which are used for PET imaging, and β^–^ particles, and Auger electrons, which are used for therapy [3, 4]. By using ^64^Cu as a radionuclide, the combination of ipRIT and PET-guided surgery can be realized with a single administration of ^64^Cu-labeled cetuximab.

In the study from Dr. Yoshii et al., the investigators tested the efficacy of ipRIT using ^64^Cu-labeled cetuximab in the first step and showed that this treatment effectively inhibited tumor growth and significantly prolonged survival with little toxicity, in mouse models with early-phase peritoneal dissemination of small lesions from gastrointestinal cancers. In the second step, they examined the feasibility of the combination use of ^64^Cu-ipRIT and PET-guided surgery using ^64^Cu-labeled cetuximab to treat late-phase peritoneal dissemination in mouse models. In this combination therapy, ^64^Cu-ipRIT is used for downstaging by treating small lesions, and OpenPET-guided surgery is used for resecting large tumor masses. The authors demonstrated that the combination use of ^64^Cu-ipRIT and OpenPET-guided surgery effectively inhibited tumor growth and significantly prolonged survival without major toxicity in mouse models with late-phase peritoneal dissemination from gastrointestinal cancers.

Notably, to make PET-guided surgery feasible, the investigators utilized the world’s first open-typed PET system, called OpenPET, which they have developed [5]. In this system, the detectors are arranged to generate an open space for surgical procedures. Additionally, to achieve real-time PET imaging under surgery, the system equips a high-speed image reconstruction system. These outstanding technologies enable real-time PET imaging. In this proof-of-concept study with mice, a small-sized OpenPET system was used. Recently, a large-sized OpenPET system for human use has been also developed by their institute [6]. Therefore, OpenPET-guided surgery will be feasible in clinical settings in the future.

Thus far, several clinical PET studies have reported the utility of ^64^Cu-labeled agents for imaging in humans. Our group has reported that ^64^Cu-labeled trastuzumab PET is a potential noninvasive procedure for the serial identification of metastatic brain lesions in patients with HER2-positive breast cancer [7]. The study suggests that PET imaging with ^64^Cu-labeled antibody is a safe and feasible approach for outpatients. The use of ^64^Cu-labeled agents for therapy is also promising. Preclinical studies have reported the therapeutic effectiveness of ^64^Cu-labeled agents, including ^64^Cu-ATSM [8], and ^64^Cu-labeled antibodies [9]. Recently, a first-in-human study of radionuclide therapy with ^64^CuCl_2_ was performed by a group of Europe, in which they showed that the patient experienced a remarkable reduction in tumor volume without side effects [10]. These studies support the usefulness and feasibility of ^64^Cu-labeled agents in humans, for both imaging and therapeutic purposes. Based on these evidences, ^64^Cu-ipRIT and PET-guided surgery, proposed by Dr. Yoshii et al., are also worthwhile for further preclinical and clinical development.
